# Trends and Characteristics of Potentially Preventable Emergency Department Visits Among Patients With Cancer in the US

**DOI:** 10.1001/jamanetworkopen.2022.50423

**Published:** 2023-01-19

**Authors:** Amir Alishahi Tabriz, Kea Turner, Young-Rock Hong, Sara Gheytasvand, Benjamin D. Powers, Jennifer Elston Lafata

**Affiliations:** 1 Department of Health Outcomes and Behavior, Moffitt Cancer Center, Tampa, Florida; 2 Department of Oncological Sciences, Morsani College of Medicine, University of South Florida, Tampa; 3 Department of Gastrointestinal Oncology, Moffitt Cancer Center, Tampa, Florida; 4 Department of Health Services Research, Management and Policy, College of Public Health and Health Professions, University of Florida, Gainesville; 5 Health Cancer Center, University of Florida, Gainesville; 6 Department of Emergency Medicine, Tabriz University of Medical Science, Tabriz, Iran; 7 Division of Pharmaceutical Outcomes and Policy, UNC Eshelman School of Pharmacy, University of North Carolina at Chapel Hill, Chapel Hill; 8 UNC Lineberger Comprehensive Cancer Center, University of North Carolina at Chapel Hill, Chapel Hill

## Abstract

**Importance:**

An initial step to reducing emergency department (ED) visits among patients with cancer is to identify the characteristics of patients visiting the ED and examine which of those visits could be prevented.

**Objective:**

To explore nationwide trends and characteristics of ED visits and examine factors associated with potentially preventable ED visits and unplanned hospitalizations among patients with cancer in the US.

**Design, Setting, and Participants:**

This cross-sectional study used data on ED visits from the National Hospital Ambulatory Medical Care Survey from January 1, 2012, to December 31, 2019; US Cancer Statistics reports were used to estimate new cancer cases each year. Frequencies and trends among 35 510 014 ED visits by adult patients (aged ≥18 years) with cancer were calculated.

**Main Outcomes and Measures:**

The primary outcome was potentially preventable ED visits, and secondary outcomes were unplanned hospitalizations and the immediacy of the ED visits. Potentially preventable ED visits were identified using the Centers for Medicare & Medicaid Services definition. The Emergency Severity Index, a triage algorithm that ranks patients based on the urgency of their health care condition, was used to measure the immediacy of ED visits (immediate [most urgent], emergent, urgent, less urgent, and nonurgent), with the categories of immediate and emergent classified as high acuity. The Wilcoxon rank sum test was used to calculate trends in ED visits among patients with cancer over time. Multivariable logistic regression analyses were performed to examine the associations of patient, hospital, and temporal factors with potentially preventable ED use and ED use resulting in hospitalization.

**Results:**

Among 854 911 106 ED visits, 35 510 014 (4.2%) were made by patients with cancer (mean [SD] age, 66.2 [16.2] years); of those, 55.2% of visits were among women, 73.2% were among non-Hispanic White individuals, 89.8% were among patients living in a private residence, and 54.3% were among Medicare enrollees. A total of 18 316 373 ED visits (51.6%) were identified as potentially preventable, and 5 770 571 visits (21.3%) were classified as high acuity. From 2012 to 2019, potentially preventable ED visits increased from 1 851 692 to 3 214 276. Pain (36.9%) was the most common reason for potentially preventable ED visits. The number of patients who visited an ED because of pain increased from 1 192 197 in 2012 to 2 405 849 in 2019 (a 101.8% increase). Overall, 28.9% of ED visits resulted in unplanned hospitalizations, which did not change significantly over time (from 32.2% in 2012 to 26.6% in 2019; *P* = .78 for trend). Factors such as residence in a nursing home (odds ratio, 1.73; 95% CI, 1.25-2.41) were positively associated with having a potentially preventable ED visit, and factors such as the presence of more than 1 comorbidity (odds ratio, 1.82; 95% CI, 1.43-2.32) were positively associated with having an unplanned hospitalization.

**Conclusions and Relevance:**

In this study, 51.6% of ED visits among patients with cancer were identified as potentially preventable, and the absolute number of potentially preventable ED visits increased substantially between 2012 and 2019. These findings highlight the need for cancer care programs to implement evidence-based interventions to better manage cancer treatment complications, such as uncontrolled pain, in outpatient and ambulatory settings.

## Key Points

**Question:**

Has there been a change in potentially preventable emergency department (ED) visits among adult patients with cancer over time?

**Findings:**

In this cross-sectional study of 35.5 million ED visits among patients with cancer, 51.6% of visits were identified as potentially preventable. From 2012 to 2019, the absolute number of potentially preventable ED visits among patients with cancer increased from approximately 1.8 million to 3.2 million.

**Meaning:**

This study’s finding of an increase in potentially preventable ED visits among patients with cancer highlights the need for cancer care programs to devise innovative ways to better manage cancer treatment complications, such as pain, in outpatient and ambulatory settings.

## Teaser

This cross-sectional study assesses trends and characteristics of potentially preventable emergency department visits and factors associated with potentially preventable visits and unplanned hospitalizations among patients with cancer in the US.

## Introduction

Patients with cancer experience numerous cancer- and treatment-related adverse effects.^1,2,3,4,5^ While many of these adverse effects can be managed in the ambulatory setting, many patients with cancer receive potentially unnecessary emergency department (ED) care due to inadequate care coordination and limited access to patient navigation and palliative care services.^6,7^ The ability to manage adverse effects in an ambulatory setting has many advantages, including improved patient experiences and the potential to avoid hospitalizations and the subsequent risk of hospital-acquired infections (eg, nosocomial pneumonia).^8,9^ In contrast, potentially unnecessary ED visits have been associated with poor patient experiences^1,2^ and increasing cancer care costs.^10,11,12^ Furthermore, EDs are often overcrowded^13^ and, because of the variety of conditions treated in the ED, patients with cancer may be exposed to communicable diseases, such as respiratory infections like influenza or COVID-19.^14,15^ Exposure to such infectious diseases is particularly detrimental to patients with cancer due to complications from cancer treatment, a damaged integumentary system, and immune system dysfunction.^16^

An initial step in reducing ED visits among patients with cancer is to identify the characteristics of ED visits among these patients and examine which visits could potentially have been prevented.^17^ Characteristics and trends of ED visits among patients with cancer have been understudied, with previous studies limited to specific types of cancer or single sites.^1,3,5,18,19,20^ Despite well-established data on potentially preventable ED visits among the general population,^21,22,23,24^ little is known about the characteristics, trends, and potential preventability of ED visits among patients with cancer. This knowledge gap has limited the ability to formulate beneficial interventions for reducing potentially unnecessary emergency care use among patients with cancer.

To address these gaps, we assessed nationwide trends and characteristics of ED visits and examined factors associated with potentially preventable ED visits and unplanned hospitalizations among patients with cancer in the US. A better understanding of such trends and the factors associated with potentially preventable visits can help clinicians and policy makers design interventions to reduce potentially unnecessary ED use among patients with cancer.

## Methods

### Study Design and Data Sources

This cross-sectional study used data from the Centers for Disease Control and Prevention (CDC) National Hospital Ambulatory Medical Care Survey (NHAMCS) from January 1, 2012, to December 31, 2019. The NHAMCS is an annual survey based on a nationally representative sample that collects information on ED use and provision of ambulatory care services from outpatient departments and ambulatory surgical centers (ASCs), short-stay and general hospitals, and freestanding ASCs. The NHAMCS uses a multistage probability design to ensure adequate representation of the hospitals, clinicians, and visits that encompass ED care in the US. Available sample weights enable estimates representative of annual ED visits nationwide.^25^ The National Center for Health Statistics provides detailed survey procedure methods.^26^ We also used data from the CDC US Cancer Statistics to estimate the new cancer cases each year.^27^ Because the data used in this study were fully deidentified and publicly available, the institutional review board of the University of North Carolina at Chapel Hill deemed the study exempt from review and the requirement for informed consent. This study followed the Strengthening the Reporting of Observational Studies in Epidemiology (STROBE) reporting guideline for cross-sectional studies.^28^

### Sample

The sample included adult patients (aged ≥18 years) with cancer who had an ED visit between 2012 and 2019. Starting in 2012, a question was added to the NHAMCS, which asks, “Does the patient have any type of cancer?”^26^ Surveyors are instructed to include only patients with current cancer diagnoses and to exclude patients with a history of cancer in remission, cancer that has been cured, or cancer diagnoses that were made during the current encounter. We used these criteria to ensure that our target population was patients with cancer who used the ED instead of patients who may have been diagnosed with cancer during an ED encounter or cancer survivors who were no longer undergoing active treatment.

### Measures

The primary outcome of interest was ED visits, including potentially preventable ED visits. There is no universally accepted definition of a potentially preventable ED visit.^4,22^ Systematic reviews^29,30^ found that 4 main approaches were used to identify potentially preventable ED visits. Some studies^29,31,32,33^ used an ED triage-based approach (eg, patient triage acuity), some used a resource use approach (eg, events that occurred during the ED visit, such as patient receipt of any medication),^21,34,35^ some used a diagnosis-based approach,^36,37^ and one used explicit criteria, such as review of nursing notes, vital signs, or duration of symptoms, as measures to define ED visits as potentially preventable.^23^ We used the Centers for Medicare & Medicaid Services (CMS) definition of a potentially preventable ED visit among patients receiving chemotherapy.^38^ The CMS defines an ED visit as potentially preventable if the primary diagnosis for the visit was one of the following: anemia, nausea, fever, dehydration, neutropenia, diarrhea, pain, pneumonia, sepsis, or emesis.^38^ To identify the primary reasons for ED visits, we used the patient’s chief concerns, symptoms, or other reasons for the ED visit. To identify the primary diagnosis associated with the current ED visit, we used the *International Classification of Diseases, Ninth Revision* for 2012 to 2015 and the *International Statistical Classification of Diseases and Related Health Problems, Tenth Revision*, for 2016 to 2019.

The secondary outcomes of interest were ED visits that resulted in hospitalization (ie, unplanned hospitalizations) and the immediacy of the ED visits. We measured the immediacy of the ED visits using the percentage of high-acuity ED visits based on the Emergency Severity Index (ESI).^39^ The ESI is a triage algorithm that ranks patients based on the urgency of their health care condition on 5 levels: (1) immediate (most urgent), (2) emergent, (3) urgent, (4) less urgent, and (5) nonurgent. The categories of immediate and emergent were classified as high acuity, consistent with a previous study.^40^ Other covariates of interest included patient-level factors, such as demographic characteristics (eg, self-reported race and ethnicity [Hispanic, non-Hispanic Black, non-Hispanic White, and other race and/or ethnicity, including American Indian or Alaska Native and Native Hawaiian or other Pacific Islander] collected from the patient’s medical record and assessed because of known disparities in ED use among different racial and ethnic groups^41^), clinical characteristics, and hospital-level factors (eg, visits by day of the week) (eTable in Supplement 1).

### Sensitivity Analyses

We conducted 2 sensitivity analyses. First, we used only unplanned admissions as the outcome because some of the ED visits may have been planned. Because planned ED visits were not directly identifiable within the NHAMCS, as a proxy, we used noninjury-related ED visits of low acuity (ESI of 4 or 5) among patients who were admitted on a weekday between 8 am and 12 am. Using this approach, we found that less than 1.0% of ED visits could be classified as planned ED admissions. Second, to mitigate possible selection bias, we used inverse probability weights to balance observed variables across both outcomes (ie, patients who were hospitalized compared with patients who were discharged and patients who visited an ED because of 10 conditions the CMS identified as potentially preventable compared with patients who visited an ED for any other reason). We then ran a logit model, weighted by propensity score, for both outcomes.

### Statistical Analysis

We addressed missing data for the outcome variables by using a multiple imputation technique^42^ and making a separate category for missing data for independent variables. Using a nonparametric test (the Wilcoxon rank sum test) for trends across ordered groups, we calculated trends in ED visits among patients with cancer over time. We used multivariable logistic regression models to test the associations of patient, hospital, and temporal factors with potentially preventable ED use and ED use resulting in hospitalization. We used Stata software, version MP 16 (StataCorp LLC), to conduct these analyses. All analyses were adjusted for the complex survey design using sampling weights based on CDC guidance (ie, adjusting for clustering and stratification). For all models, we used a significance level of 2-sided *P* = .05. Results were reported as frequencies with percentages and odds ratios (ORs) with 95% CIs.

## Results

### Trends and Characteristics of ED Visits

Between 2012 and 2019, 35 510 014 of 854 911 106 ED visits (4.2%; 95% CI, 3.6%-4.8%) were made by patients with cancer (mean [SD] age, 66.2 [16.2] years). Of those, 55.2% of visits were among women, 73.2% were non-Hispanic White individuals, and 54.3% were among Medicare enrollees. Furthermore, most of these visits were among individuals who lived in private residences (89.8%) and urban areas (84.6%) (Table 1). The percentage of ED visits among patients with cancer increased by 67.1%, from 3 734 101 visits (3.6% of all ED visits) in 2012 to 6 240 650 visits (5.4% of all ED visits) in 2019 (*P* < .001 for trend). The percentage of ED visits made by patients with cancer per new cancer case increased by 45.1%, from 2.44 visits in 2012 to 3.54 visits in 2019.

**Table 1.** Characteristics of Emergency Department Visits Among Adult Patients With Cancer in the US, 2012-2019

Characteristic	ED visits, No. (%)	
	Total weighted (N = 35 510 014)	Potentially preventable (n = 18 316 373)
Sex		
Male	15 898 094 (44.8)	7 711 620 (42.1)
Female	19 611 920 (55.2)	10 604 753 (57.9)
Race and ethnicity		
Hispanic	3 214 174 (9.0)	1 719 152 (9.4)
Non-Hispanic		
Black	5 323 598 (15.0)	2 986 153 (16.3)
White	25 979 008 (73.2)	13 100 672 (71.5)
Other^a^	993 233 (2.8)	510 396 (2.8)
Residence		
Private	31 905 264 (89.8)	16 882 575 (92.2)
Nursing home	1 707 655 (4.8)	603 960 (3.3)
Homeless or other	802 089 (2.3)	351 496 (1.9)
Missing	1 095 004 (3.1)	478 341 (2.6)
Primary payment type		
Private insurance	6 584 417 (18.5)	3 840 593 (21.0)
Medicare	19 296 103 (54.3)	9 548 445 (52.1)
Medicaid	4 671 037 (13.2)	2 619 592 (14.3)
Self-payment	1 045 912 (2.9)	491 079 (2.7)
Other	811 024 (2.3)	367 297 (2.0)
Missing	3 101 519 (8.7)	1 449 367 (7.9)
Hospital disposition		
Admitted	10 252 316 (28.9)	5 528 390 (30.2)
Discharged	25 257 697 (71.1)	12 787 983 (69.8)
Triage acuity		
Immediate	349 224 (1.0)	119 773 (0.6)
Emergent	5 421 347 (15.3)	2 853 315 (15.6)
Urgent	14 402 994 (40.6)	7 916 459 (43.2)
Semiurgent	4 346 389 (12.2)	2 048 581 (11.2)
Nonurgent	574 208 (1.6)	161 940 (0.9)
No triage^b^	2 142 459 (6.0)	1 101 033 (6.0)
Missing	8 273 392 (23.3)	4 115 271 (22.5)
Region		
Northeast	6 174 030 (17.4)	3 004 528 (16.4)
Midwest	9 446 297 (26.6)	4 615 891 (25.2)
South	12 755 647 (35.9)	6 909 595 (37.7)
West	7 134 039 (20.1)	3 786 359 (20.7)
Urbanity^c^		
MSA	26 892 983 (84.6)	14 053 872 (85.3)
Non-MSA	4 882 930 (15.4)	2 415 722 (14.7)
Day of week		
Weekday	25 866 230 (72.8)	13 190 496 (72.0)
Weekend	9 643 783 (27.2)	5 125 877 (28.0)

Abbreviations: ED, emergency department; MSA, metropolitan statistical area.

^a^ Other races and ethnicities include American Indian or Alaska Native and Native Hawaiian or other Pacific Islander.

^b^ Visits in which the emergency service area coded the nursing triage as 0 (admitted to hospital or treated immediately) or visits that occurred in an emergency service area that did not conduct triage.

^c^ Data for 2012 were not available. Therefore, total weighted percentages were based on 31 775 913 ED visits, and percentages for potentially preventable percentages were based on 16 469 594 ED visits.

### Trends and Characteristics of Potentially Preventable ED Visits

A total of 18 316 373 ED visits (51.6%) by patients with cancer were identified as potentially preventable. Between 2012 and 2019, the percentage of potentially preventable ED visits among patients with cancer did not change significantly (from 49.6% in 2012 to 51.5% in 2019; *P* = .11 for trend) (Figure). However, the absolute number of potentially preventable ED visits (from 1 851 692 visits in 2012 to 3 214 276 visits in 2019; 73.6% increase) and potentially preventable ED visits per new cancer case (from 1.2 visits in 2012 to 1.8 visits in 2019) increased significantly (for both comparisons, *P* < .001 for trend).

**Figure.**
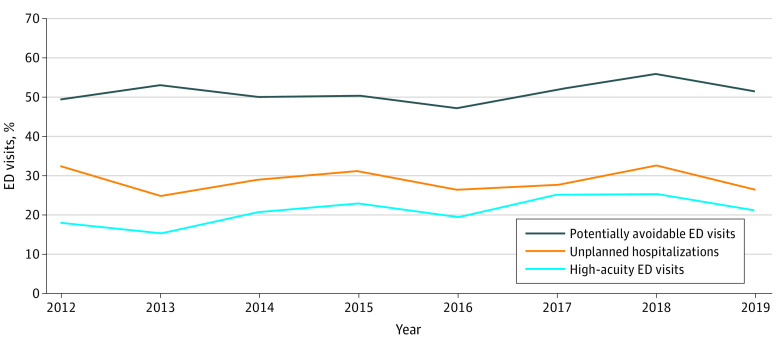
Trends in the Percentage of Emergency Department (ED) Visits Among US Patients With Cancer, 2012-2019 Among 35 510 014 ED visits by patients with cancer.

Of the 10 conditions the CMS considered to be potentially preventable reasons for ED visits, pain (36.9%) was the most common primary complaint, followed by fever (3.5%), nausea (3.5%), and emesis (2.3%) (Table 2). The number of patients who visited an ED because of pain increased from 1 192 197 in 2012 to 2 405 849 in 2019 (101.8% increase; *P* < .001 for trend). Furthermore, we found pain was the diagnosis code for 39.8% of ED visits that resulted in discharge from the ED and 33.1% of high-acuity ED visits. Similar increases were observed for other potentially preventable conditions (except for emesis and pneumonia).

**Table 2.** Frequencies and Trends for the Top 15 Conditions Cited as Reasons for Emergency Department Visits Among Adult Patients With Cancer in the US, 2012-2019

Condition	Weighted frequency			Percentage change between 2012 and 2019^a^
	Total visits, No. (%) (N = 35 510 014)	Visits in 2012, No.	Visits in 2019, No.	
Pain^b^	13 113 594 (36.9)	1 192 197	2 405 849	101.8
Fever^b^	1 231 595 (3.5)	108 980	192 617	76.8
Nausea^b^	1 230 072 (3.5)	126 328	139 208	10.2
Emesis^b^	825 200 (2.3)	176 023	116 687	−33.7
Dyspnea	654 214 (1.8)	73 590	71 213	−3.2
Pneumonia^b^	616 028 (1.7)	88 568	84 959	−4.1
UTI	486 913 (1.4)	54 953	73 161	33.1
Fatigue	482 790 (1.4)	73 119	102 758	40.5
Diarrhea^b^	445 522 (1.3)	56 708	90 974	60.4
COPD with acute exacerbation	423 750 (1.2)	26 729	112 832	322.1
Syncope and collapse	405 116 (1.1)	41 407	50 825	22.7
Dehydration^b^	339 850 (1.0)	28 509	82 601	189.7
Dizziness and giddiness	276 355 (0.8)	38 449	46 837	21.8
Anemia^b^	237 940 (0.7)	26 398	48 176	82.5
Sepsis^b^	220 072 (0.6)	27 584	53 201	92.9

Abbreviations: COPD, chronic obstructive pulmonary disease; UTI, urinary tract infection.

^a^ *P *< .001 for all.

^b^ Conditions that were categorized by the Centers for Medicare & Medicaid Services as potentially preventable.

### Trends and Characteristics of High-Acuity ED Visits and Unplanned Hospitalizations

The ESI for 23.3% of ED visits was not reported, and for 6.0% of visits, triage was not completed (ie, patients were admitted to the hospital or treated immediately, or the visit occurred in an ED that did not conduct triage). Among ED visits with a reported ESI, 5 770 571 (21.3%) were classified as immediate (1.3%) or emergent (20.0%) and considered high acuity. Between 2012 and 2019, the percentage of patients with cancer who visited the ED with high acuity increased by 15.8% (from 18.3% in 2012 to 21.1% in 2019; *P* < .001 for trend). Between 2012 and 2019, high-acuity cancer-related ED visits per new cancer case increased by 71.0%, from 0.31 visits to 0.53 visits.

We found that 28.9% of ED visits among patients with cancer resulted in unplanned hospitalizations. Between 2012 and 2019, the percentage of patients with cancer who visited the ED and were admitted to an inpatient unit did not change significantly (from 32.2% in 2012 to 26.6% in 2019; *P* = .78 for trend); however, hospital admissions per new cancer case increased by 21.9%, from 0.73 admissions to 0.89 admissions. Overall, among ED visits that were considered potentially preventable by the CMS, we found that 30.2% resulted in hospitalization. Furthermore, we found a wide range of hospitalization rates among the 10 conditions the CMS identified as potentially preventable reasons for ED visits. Sepsis (93.3%) had the highest admission rate, followed by pneumonia (76.2%) and anemia (71.7%), while pain (23.5%), dehydration (28.0%), and nausea (31.8%) had the lowest hospitalization rates.

### Factors Associated With Potentially Preventable ED Visits and Unplanned Hospitalizations

Factors such as male sex (OR, 1.19; 95% CI, 1.04-1.36) and residence in a nursing home (OR, 1.73; 95% CI, 1.25-2.41) were positively associated with having a potentially preventable ED visit (Table 3). Conversely, factors such as non-Hispanic Black race (OR, 0.83; 95% CI, 0.69-0.99) and age younger than 65 years (OR, 0.73; 95% CI, 0.61-0.87) were negatively associated with having a potentially preventable ED visit.

**Table 3.** Association Between Patient Characteristics and Potentially Preventable Emergency Department Visits and Unplanned Hospitalizations Among Adult Patients With Cancer in the US, 2012-2019^a^

Characteristic	OR (95% CI)	
	Unplanned hospitalizations	Potentially preventable ED visits
Age, y		
18-64	0.73 (0.60-0.88)	0.73 (0.61-0.87)
≥65	1 [Reference]	1 [Reference]
Sex		
Female	1 [Reference]	1 [Reference]
Male	1.16 (1.00-1.34)	1.19 (1.04-1.36)
Race and ethnicity		
Hispanic	1.57 (1.11-2.22)	0.94 (0.73-1.22)
Non-Hispanic Black	1.14 (0.89-1.44)	0.83 (0.69-0.99)
Non-Hispanic White	1 [Reference]	1 [Reference]
Other^b^	1.25 (0.73-2.14)	1.00 (0.65-1.54)
Residence		
Private	1 [Reference]	1 [Reference]
Nursing home	1.33 (0.85-2.06)	1.73 (1.25-2.41)
Homeless or other	1.43 (0.82-2.49)	1.29 (0.80-2.08)
Missing	1.21 (0.79-1.83)	1.33 (0.96-1.84)
Visit acuity		
High	1 [Reference]	NA
Low	0.35 (0.28-0.45)	NA
No triage	0.47 (0.28-0.80)	NA
Missing	0.45 (0.33-0.63)	NA
Potential preventability		NA
Potentially preventable ED visits	1 [Reference]	NA
Necessary ED visits	0.79 (0.67-0.90)	NA
No. of comorbidities		
0	1 [Reference]	1 [Reference]
1	1.29 (0.98-1.71)	0.89 (0.69-1.17)
>1	1.82 (1.43-2.32)	0.91 (0.70-1.23)
Primary payment type		
No insurance	1 [Reference]	1 [Reference]
Medicare	1.56 (0.78-3.11)	0.61 (0.36-1.02)
Medicaid	0.94 (0.45-1.97)	0.63 (0.37-1.08)
Private insurance	1.34 (0.63-2.87)	0.57 (0.34-0.96)
Other	0.98 (0.39-2.48)	0.92 (0.46-1.83)
Missing	0.79 (0.35-1.76)	0.79 (0.46-1.37)
Urbanity^c^		
MSA	1 [Reference]	1 [Reference]
Non-MSA	0.35 (0.23-0.53)	0.19 (0.82-1.27)
Region		
Midwest	1 [Reference]	1 [Reference]
Northeast	1.10 (0.73-1.66)	1.01 (0.81-1.27)
South	0.75 (0.49-1.17)	0.84 (0.73-0.97)
West	1.06 (0.71-1.58)	0.87 (0.68-1.12)
Day of week		
Weekday	1 [Reference]	NA
Weekend	0.93 (0.76-1.12)	NA
Patient arrival time		
12 am to <8 am	1 [Reference]	NA
8 am to <4 pm	1.18 (0.92-1.52)	NA
4 pm to <12 am	1.37 (0.99-1.75)	NA

Abbreviations: ED, emergency department; MSA, metropolitan statistical area; NA, not applicable; OR, odds ratio.

^a^ Among 35 510 014 total ED visits.

^b^ Other races and ethnicities include American Indian or Alaska Native and Native Hawaiian or other Pacific Islander.

^c^ Data for 2012 were not available. Data are based on 25 535 262 ED visits and hospitalizations.

Factors such as Hispanic ethnicity (OR, 1.57; 95% CI, 1.11-2.22) and the presence of more than 1 comorbidity (OR, 1.82; 95% CI, 1.43-2.32) were positively associated with unplanned hospitalization. Conversely, factors such as low-acuity ED visits (OR, 0.35; 95% CI, 0.28-0.45) and age younger than 65 years (OR, 0.73; 95% CI, 0.60-0.88) were negatively associated with unplanned hospitalization (Table 3). The results of both sensitivity analyses were not significantly different from the results reported in this article.

## Discussion

We conducted this cross-sectional study to explore nationwide trends and characteristics of ED visits (including the main reasons for ED presentation) among patients with cancer and examine factors associated with potentially preventable ED visits and unplanned hospitalizations. Consistent with previous studies,^43,44^ we found that nationwide each year, 4.2% of all ED visits were made by patients with cancer. This rate was similar to the rate of ED visits reported for other conditions, such as congestive heart failure (4.0%), chronic kidney disease (3.5%), and cerebrovascular disease (3.7%).^45^ We also found that between 2012 and 2019, the number of cancer-related ED visits increased by 67.1% compared with cancer incidence, which only increased by 7.5%.^46,47^ The disproportionate increase in the number of ED visits by patients with cancer has put a substantial burden on EDs that are already operating at peak capacity.^48,49^ Several factors may explain this finding, including the aging population,^50,51^ the availability of novel therapy options,^52^ the increasing use of oral or topical chemotherapy,^53^ and the general shift from inpatient to ambulatory cancer care.^54,55^ Because EDs are generally not an optimal setting to provide care for patients with medically complex cancer, this increase in ED visits among patients with cancer reinforces the need for cancer care programs to devise innovative ways to manage complications associated with cancer treatment in the outpatient and ambulatory settings.^56^

We found that the rate of potentially preventable ED visits among patients with cancer was 51.6%, which was higher than the rate reported in previous studies.^3,57,58^ This difference may be because a nationwide sample was used (eg, rates in population-based studies are generally higher than those in small single-setting studies)^4^ or the way in which potentially preventable ED visits (using CMS criteria) were classified. We also found that the absolute number of potentially preventable ED visits increased by 73.6%, largely because of the significant increase in patients with cancer who visited the ED because of uncontrolled pain.

Consistent with previous studies,^1,2,3,5,6,18^ we found that pain was the most common presenting symptom (36.9%) in ED visits among patients with cancer and that the number of patients with cancer who visited an ED because of pain more than doubled over the study period. A possible explanation could be the unintended consequences of the efforts within the past decade to decrease overall opioid administration in response to the opioid epidemic.^59,60^ For example, a previous study^5^ found that about half of patients with cancer who had severe pain did not receive outpatient opioids in the week before an ED visit occurred. Similarly, a study at MD Anderson Cancer Center reported that between 2010 and 2015, the number of opioid prescriptions by referring oncologists decreased substantially.^61^ There is a need to develop and test new interventions that provide oncologists with the necessary training in providing beneficial pain management (eg, pharmacological and nonpharmacological) while maintaining adequate safeguards to prevent opioid abuse.^62^

We found that other conditions, such as fever, nausea, emesis, dyspnea, fatigue, and urinary tract infections, were among the most common presenting symptoms in a patient with cancer. Of those conditions, dyspnea, urinary tract infections, fatigue, chronic obstructive pulmonary disease with acute exacerbation, syncope and collapse, and dizziness and giddiness were not among the conditions that the CMS considered to be potentially preventable reasons for ED visits. Because previous research^2,44^ identified conditions such as fatigue or urinary tract infections as common reasons for ED visits in patients with cancer and because these conditions can be effectively managed in an outpatient setting, they may be considered potentially preventable reasons for ED visits.

We found that 21.3% of ED visits by patients with cancer could be categorized as high acuity, which is higher than the percentage of high-acuity ED visits among the general population.^63^ This finding was consistent with previous studies^39,45^ that found the ESI distribution of patients with cancer was of substantially higher acuity than that of the general population. Higher acuity at the time of an ED visit could be explained by complications of cancer treatment and comorbidities or by the way the ESI triage algorithm was designed.^64^ Based on the ESI triage algorithm, if a patient was in a high-risk situation at ED presentation, the patient would be triaged as ESI level 1 or level 2 (ie, high acuity). A previous study^39^ found that the ESI is a valid triage tool for use in populations with cancer. With the ESI, triage staff use the patient's medical history and presenting symptoms to determine acuity. Therefore, the triage nurses categorize patients with cancer as high acuity compared with patients without cancer who have otherwise similar presentations because patients with cancer have a higher risk of developing complications compared with patients without cancer. For example, based on the ESI triage algorithm, a patient with a fever who is receiving chemotherapy should be triaged as ESI level 2 (because of a substantially higher risk of neutropenic fever), while a patient without cancer who has a fever might be triaged as lower acuity.^64^

We found that 28.9% of ED visits among patients with cancer resulted in unplanned hospitalizations. Previous studies^1,2,44,65^ reported higher rates of hospital admission among patients with cancer. The lower rate of unplanned hospitalizations in this study could be because of the NHAMCS sampling methods (ie, the NHAMCS contains low-volume and low-acuity EDs and freestanding ASCs).^13^

Consistent with a previous study,^64^ we found that acuity at the time of ED presentation was associated with hospitalization. However, although the percentage of high-acuity ED visits increased during the study period, we found that the hospitalization rate among patients with cancer did not change significantly over time. We compared high-acuity ED visits and ED visits that resulted in hospitalization to examine how their profiles differed. The key difference between these 2 groups was their ED diagnosis codes. While pain was the ED diagnosis code for 39.8% of ED visits that did not result in hospitalization, it was the ED diagnosis code for 33.1% of high-acuity ED visits. The significant increase (101%) in the number of patients with cancer who visited an ED because of pain may explain the decrease in hospitalization rate and the increase in high-acuity ED visits over time. This finding may imply that while many patients with cancer visited high-acuity EDs because they were experiencing pain, they were discharged when their pain was controlled at the ED, with no need for further admission. This finding also emphasized the importance of pain management in the outpatient setting.

We found that among ED visits that were considered potentially preventable by the CMS, 30.2% resulted in hospitalization. This finding implies that if symptom management had been better from the start or if timely and beneficial ambulatory care had been available, those patients would not have gotten to the point at which they needed to visit the ED. However, when conditions such as pneumonia or sepsis occurred, ED visits were warranted. For example, we found that 93.3% of patients who visited EDs with sepsis and 76.2% of patients who visited EDs with pneumonia were hospitalized. Both sepsis and pneumonia can be prevented with proper postdischarge care (eg, remote symptom management).

It is notable that the risks, inconveniences, and costs associated with ED visits among patients with cancer did not seem to be borne evenly across the population with cancer. For example, factors such as living in a nursing home were positively associated with having a potentially preventable ED visit. This finding may be explained by a practice pattern; a previous study^66^ reported that most referrals from nursing homes to the ED were potentially preventable. We also found that non-Hispanic Black patients with cancer were less likely to visit the ED for potentially preventable reasons. This behavior may be explained by factors such as known racial disparities in health care that discourage them from seeking care for reasons like pain; many studies included in a meta-analysis^67^ found that Black patients were less likely to receive medication to control their pain compared with White patients.

We also found that Hispanic ethnicity, age older than 65 years, and the presence of more comorbidities were associated with significant increases in the likelihood of hospitalization when a patient with cancer presented to the ED. These findings were consistent with those of previous studies^57,68,69^ and may be reflective of a lack of access to care, inadequate social support, poor comorbidity management, or a combination of these and other factors. As such, the findings again highlighted the important role that nonclinical variables, such as social factors associated with health and access to appropriate services, play in the use of health care services.^70,71^

### Limitations

This study has several limitations. First, because some information is not collected by the NHAMCS, we were unable to consider other important factors, such as cancer type and stage, cancer treatment type and duration, usual source of care, or level of social support (eg, caregiver). Second, we used the CMS definition of potentially preventable ED visits that was designed for use with patients receiving chemotherapy. The applicability of this classification to patients with cancer who are receiving other treatments (eg, radiotherapy or surgical procedures) is not known. Third, propensity score methods account for selection bias based on observed factors; however, we still cannot account for unobservable factors that may be associated with potentially preventable ED visits and unplanned hospitalizations. Fourth, given changes in practice patterns (eg, telehealth oncology programs^72^) and patient care-seeking behavior due to the COVID-19 pandemic, studies are needed to assess the implications of the COVID-19 pandemic for ED visits among patients with cancer.

## Conclusions

In this cross-sectional study of ED visits among adult patients with cancer in the US, the number of potentially preventable ED visits increased over time, which may be explained by poorly managed symptoms, such as uncontrolled pain. These findings reinforce the need for cancer care programs to implement evidence-based interventions to better manage cancer treatment complications, such as pain, in outpatient and ambulatory settings.

## Notes

Supplement 1. **eTable.** All Variables Included in the Study

Supplement 2.
Data Sharing Statement
