# Supplementary material for: Trends and Characteristics of Potentially Preventable Emergency Department Visits Among Patients With Cancer in the US
Source: JAMA Netw Open. 2023 Jan 19;6(1):e2250423. doi: 10.1001/jamanetworkopen.2022.50423 (PMC9857289; doi:10.1001/jamanetworkopen.2022.50423)
Supplement: Supplement 1. — eTable. All Variables Included in the Study [file jamanetwopen-e2250423-s001.pdf]

Supplemental Online Content

Alishahi Tabriz A, Turner K, Hong YR, Gheytasvand S, Powers BD, Elston Lafata J. Trends and characteristics of potentially preventable emergency department visits among patients with cancer in the US. *JAMA Netw Open*. 2023;6(1):e2250423. doi:10.1001/jamanetworkopen.2022.50423

eTable. All Variables Included in the Study

This supplemental material has been provided by the authors to give readers additional information about their work.

© 2023 Tabriz AA et al. *JAMA Network Open*.

eTable. All Variables Included in the Study

Name	Description
**Outcome variable**	
Potentially preventable ED visit (based on CMS-OP35)[1]	Any ED visit due to one of these eight conditions will be considered as potentially avoidable; 1- Anemia, 2- Nausea, 3- Dehydration, 4- Neutropenia, 5- Diarrhea, 6- Pain, 7- Fever, 8- Emesis, 9- Sepsis, 10- Pneumonia.
**Main Independent variable**	
If patient already diagnosed with cancer	Yes, No, Missing
**Patient level variables (control variables)**	
Patient age	18-64 (Adults), ≥ 65 (Seniors), Missing
Patient sex	Male, Female, Missing
Patient race and ethnicity (Self-reported)	Withe non-Hispanic, Black non-Hispanic, Hispanic, Other
Patient residence	Private residence, Nursing home, Other residence, Missing
Patient primary payment type	Private insurance, Medicare, Medicaid, Uninsured/self pay, Missing
Patient triage acuity (the immediacy by which a patient needed to be seen by a physician)	Immediate (15 minutes), Emergent (15–60 minutes), Urgent (>1–2 hours), Semi-urgent (>2–24 hours), Non-urgent (more than 24 hours), No-triage, Missing
Total number of chronic conditions	0, 1, 2, 3, more than 3
Patient disposition	Admitted to hospital, discharged, Missing
**Hospital level factors (control variables)**	
Region	Northeast, Midwest, South, West, Missing
Urbanity	Metropolitan statistical area (MSA), Non-MSA, Missing
**Temporal factor (control variables)**	
Patient arrival time	Midnight-8am, 8am-4pm, 4pm-midnight, Missing
Day of week	Weekend, Weekday
Month of the year	January, February, March, April, May, June, July, August, September, October, November, and December.

1. Services C for M& M. Admissions and Emergency Department Visits for Patients Receiving Outpatient Chemotherapy Measure Technical Report.; 2018.

© 2023 Tabriz AA et al. *JAMA Network Open*.
