# Supplementary material for: Trends and Characteristics of Potentially Preventable Emergency Department Visits Among Patients With Cancer in the US
Source: JAMA Netw Open. 2023 Jan 19;6(1):e2250423. doi: 10.1001/jamanetworkopen.2022.50423 (PMC9857289; doi:10.1001/jamanetworkopen.2022.50423)
Supplement: Supplement 2. — Data Sharing Statement [file jamanetwopen-e2250423-s002.pdf]

Data Sharing Statement

Alishahi Tabriz. Trends and Characteristics of Potentially Preventable Emergency Department Visits Among Patients With Cancer in the US. *JAMA Netw Open*. Published January 19, 2023. doi:10.1001/jamanetworkopen.2022.50423

Data
Data available: Yes
Data types: Deidentified participant data
How to access data: https://www.cdc.gov/nchs/ahcd/datasets_documentation_related.htm
When available: With publication

Supporting Documents
Document types: None

Additional Information
Who can access the data: Public.
Types of analyses: For any purpose.
Mechanisms of data availability: Without investigator support
